# Determination of Alkaloids and Flavonoids in *Sophora flavescens* by UHPLC-Q-TOF/MS

**DOI:** 10.1155/2021/9915027

**Published:** 2021-07-28

**Authors:** Yaqian Dong, Guoxiang Jia, Jingwen Hu, Hui Liu, Tingting Wu, Shenshen Yang, Yubo Li, Ting Cai

**Affiliations:** ^1^School of Traditional Chinese Materia Medica, Tianjin University of Traditional Chinese Medicine, No. 10 Poyang Lake Road, Tuanbo New City, Jinghai District, Tianjin 301617, China; ^2^Hwa Mei Hospital, University of Chinese Academy of Sciences (Ningbo No. 2 Hospital), Ningbo 315010, China; ^3^Ningbo Institute of Life and Health Industry, University of Chinese Academy of Sciences, Ningbo 315010, China

## Abstract

This study is based on UHPLC-Q-TOF/MS and fragment ions to achieve classification and identification of alkaloids and flavonoids in *Sophora flavescens*. By reviewing the available and relevant literature, the mass fragmentation rules of alkaloids and flavonoids were summarized. 0.1% formic acid water (A) and acetonitrile (B) were used as mobile phases. 37 chemical constituents were identified, including 13 alkaloids and 24 flavonoids. This research method offers a complete strategy based on the fragmentation information of characteristic fragment ions and neutral loss obtained by MS/MS to characterize the chemical composition of *Sophora flavescens*.

## 1. Introduction

The analytical methods of traditional Chinese medicine (TCM) are not sufficient for the separation and identification of many complex chemical components, which brings challenges in terms of the quality control and clinical application of TCM [1]. Ultrahigh-performance liquid chromatography-quadrupole time-of-flight mass spectrometry (UHPLC-Q-TOF/MS) has become the main means of component analysis of modern traditional Chinese medicine because of its high speed, high efficiency, and high resolution. It can overcome the limitation of ultraviolet detectors, so it is suitable for component analysis in the complex traditional Chinese medicine system [2–4]. The chemical components in TCM can be classified and quickly identified on the basis of secondary fragments [5, 6]. In the process of treating diseases, traditional Chinese medicine often has multiple components and multiple targets, which often lead to the problem of unclear components. Therefore, the classification and identification of chemical components in traditional Chinese medicine are very meaningful. According to differences in the chemical structure, the compounds can be divided into different parent nuclear structure types. Compounds with the same parent nuclear type will produce some ion fragments which are the same in the process of mass spectrometry collision.

The traditional Chinese medicinal herb *Sophora flavescens* comes from dried roots of *Sophora flavescens* Ait., a leguminous plant which is listed as middle grade in Shennong Materia Medica, and is bitter and cold in taste. Alkaloids and flavonoids are considered to be the main active components of *Sophora flavescens* [7]. Studies have shown that alkaloids in *Sophora flavescens* can reduce the secretion of inflammatory factor TNF-*α* by regulating the expression of BMP2, Runx2, and other proteins, so as to increase the activity of alkaline phosphatase to treat chronic osteomyelitis caused by *Staphylococcus aureus* infection [8]. Indoleamine 2-dioxygenase-1 (IDO1), a tumor cell survival factor, can lead to the escape of many kinds of cancer cells. As inhibitors of IDO1, many flavonoids in *Sophora flavescens* have potential uses in cancer immunotherapy [9]. In view of the good clinical efficacy and research prospects of *Sophora flavescens*, it is of great significance to establish a technique that can quickly classify and identify the chemical composition of *Sophora flavescens*.

Based on the UHPLC-Q-TOF/MS technology, this study summarized the characteristic fragments and neutral losses during the cleavage process of compounds, classified and identified the chemical components in *Sophora flavescens*, and identified 37 alkaloids and flavonoids in *Sophora flavescens*.

## 2. Methods

### 2.1. Materials and Instruments

*Sophora flavescens* (Beijing Tongren Drug Store), matrine, oxymatrine, sophocarpine standard (Chengdu Ruifensi Biotechnology Co., Ltd., China), ethanol (Tianjin Huihang, analytical grade), acetonitrile (Sigma, USA, HPLC grade), formic acid (Sigma, USA, HPLC grade), distilled water (Guangzhou Watsons, China), UPLC-Q-TOF-MS (Waters, Milford, MA, USA), a Waters ACQUITY UPLC BEH C18 column (100 mm × 2.1 mm, 1.7 *μ*m), and MassLynx V4.1 were used.

### 2.2. Preparation of Samples

5.0 g of *Sophora flavescens* was precisely weighed, refluxed, and extracted twice with 8 times and 6 times of 70% ethanol for 2 hours each time. The combined extract was evaporated and concentrated to 0.1 g/mL and then filtered by a 0.22 *μ*m microporous membrane, which was the sample solution to be injected [10, 11].

1 mg of matrine, oxymatrine, and sophocarpine was precisely weighed. Then, 1 ml of 70% ethanol was added to dissolve and passed through a 0.22 *μ*m microporous filter membrane.

### 2.3. UHPLC and MS Conditions

  UHPLC: Waters ACQUITY UHPLC BEH C18 Column, 2.1 × 100 mm, 1.7 *μ*m; column temperature is set to 35°C; mobile phase: the aqueous phase is 0.1% formic acid aqueous solution (A), and the organic phase is acetonitrile solution (B); flow rate: 0.4 mL/min. The gradient elution method is used for chromatographic separation, and the gradient procedure is as follows: 0–10 min, 3–20% B; 10–15 min, 20–30% B; 15–20 min, 30–50% B; 20–25 min, 50–70% B; 25–27 min, 70–100% B; 27–30 min, 100% B; 30–32 min,100–3% B; 32–35 min, 3% B
  TOF-MS: electrospray ionization source (ESI), scanning mode: positive and negative ions. The MS parameters are as follows: dry gas temperature: 325°C; dry gas flow rate: 11 ml/min; desolvent gas flow rate: 800 L/h; capillary voltage: 3.0 kV; collision-induced dissociation voltage: 6 kV; collision energy: 20–50 eV; atomizer pressure: 350 psi; auxiliary gas: N_2_; positive and negative reference ion calibration ([M+H]^+^ = 556.2771, [M-H]^−^ = 554.2615) to ensure accuracy in spectral acquisition. The range of data acquisition is 50 to 1500.

## 3. Results and Discussion

### 3.1. Establishment of the Method

**Figure 1:**
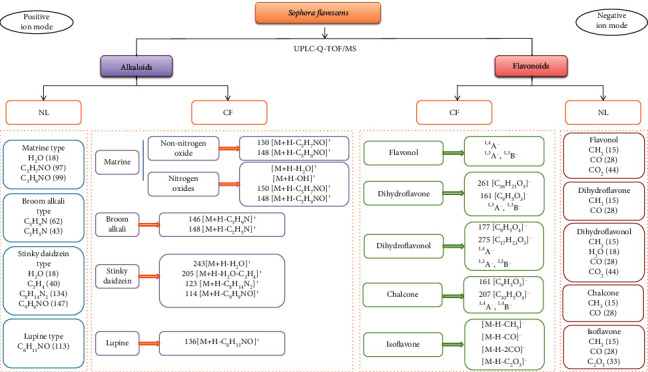
Characteristic fragments and neutral loss of the chemical composition of *Sophora flavescens*.

**Figure 2:**
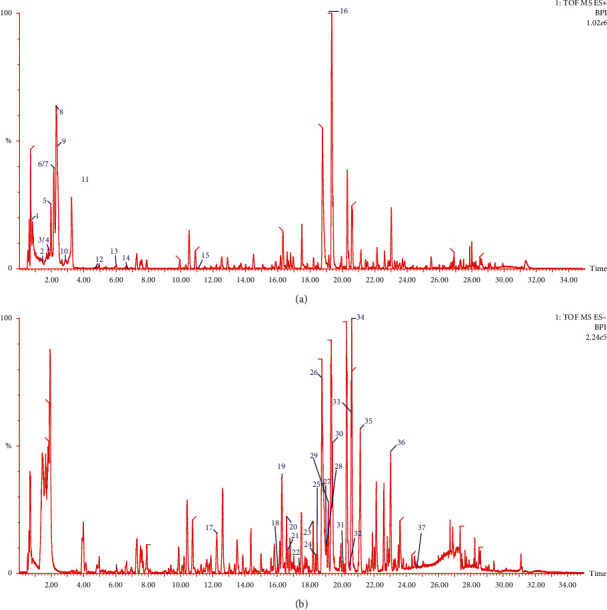
The base peak ion (BPI) flow diagram of *Sophora flavescens* in the (a) positive and (b) negative mode.

The mass spectrometry experimental data reported in the literature were used to summarize the fragments missing from the fragment ion peaks of known chemical components in *Sophora flavescens* and summarize the fragmentation rules of different fragment ions. Subsequently, MassLynx software was used for peak matching, and the chemical composition of *Sophora flavescens* was deduced based on the retention time of its components and the fragmentation rules. Finally, 13 alkaloids and 24 flavonoids were identified, as shown in Table 1. The fragmentation rules of the chemical components in *Sophora flavescens* are shown in Figure 1, and the base peak ion (BPI) chromatogram of the *Sophora flavescens* extract in positive and negative ions is shown in Figure 2.

### 3.2. Fragmentation Rules of Alkaloid Compounds

According to the structure type of the mother nucleus, the alkaloids in *Sophora flavescens* are mainly divided into matrine type, broom alkali type, anagyrine type, and lupine type [25]. Among them, matrine-type compounds easily lose H_2_O (18), C_5_H_7_NO (97), and C_5_H_9_NO (99) in the collision process, resulting in characteristic fragments of *m/z* 150 and *m/z* 148. Nitrogen oxides of matrine alkaloids easily lose H_2_O (18) and OH (17), resulting in high-abundance fragments [M+H-H_2_O]^+^ and [M+H-OH]^+^ [13, 14]. The cleavage of C7-C13/C9-C11 and C6-C7/C1-C10 of broom alkaloid bonds will produce characteristic fragments such as 146[M+H-C_3_H_9_N]^+^ and 148[M+H-C_2_H_5_N]^+^ which are related to the methyl substituents at position 12 [12]. The characteristic fragments of daidzein alkaloids include 243[M+H-H_2_O]^+^, 205 [M+H-H_2_O-C_3_H_4_]^+^, 123[M+H-C_8_H_14_N_2_]^+^, and 114[M+H-C_9_H_8_NO]^+^ [17].

**Figure 3:**
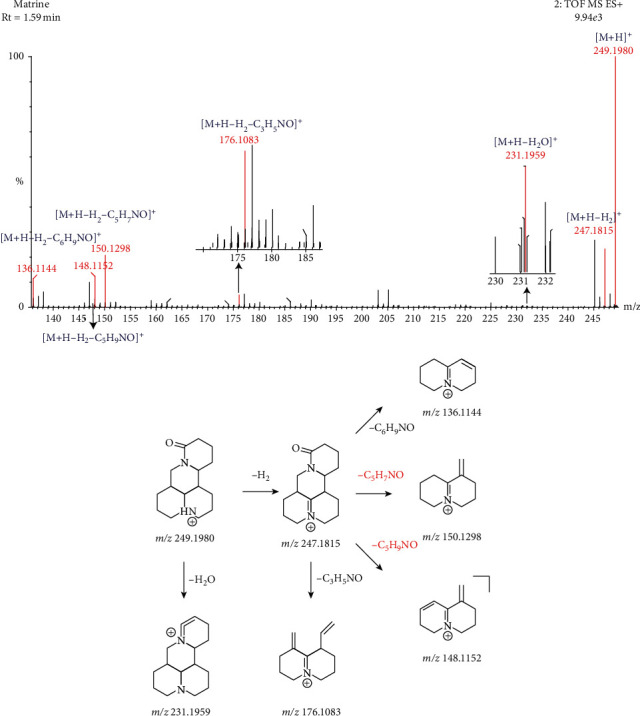
The fragmentation process of matrine in the positive ion mode.

The molecular formula of compound 2 is C_15_H_24_N_2_O, and the retention time is 1.59 min. The main secondary fragments are 247.1815, 231.1959, 176.1083, 150.1298, 148.1152, and 136.1144. In the positive ion mode, the molecular ion peak is *m/z* 249.1980[M+H]^+^, and its parent ion removes a molecule of H_2_ and H_2_O, respectively, resulting in an ion peak of *m/z* 247.1815[M+H-H_2_]^+^and a dehydration peak of *m/z* 231.1959[M+H-H_2_O]^+^. Then, the compound undergoes RDA cleavage. On this basis, characteristic matrine-type ion fragments are *m/z* 150.1298[M+H-H_2_-C_5_H_7_NO]^+^, *m/z* 148.1152[M+H-H_2_-C_5_H_9_NO]^+^, and *m/z* 136.1144 [M+H-H_2_-C_6_H_9_NO]^+^. On the basis of losing a molecule of H_2_, the compound can continue to lose a molecule of C_3_H_5_NO and form an ion peak of *m/z* 176.1083[M+H-H_2_-C_3_H_5_NO]^+^. According to fragment information, standard reference substance retention time, relative molecular mass, and MS and MS information, the compound is identified as matrine. The fragmentation rules are shown in Figure 3.

**Figure 4:**
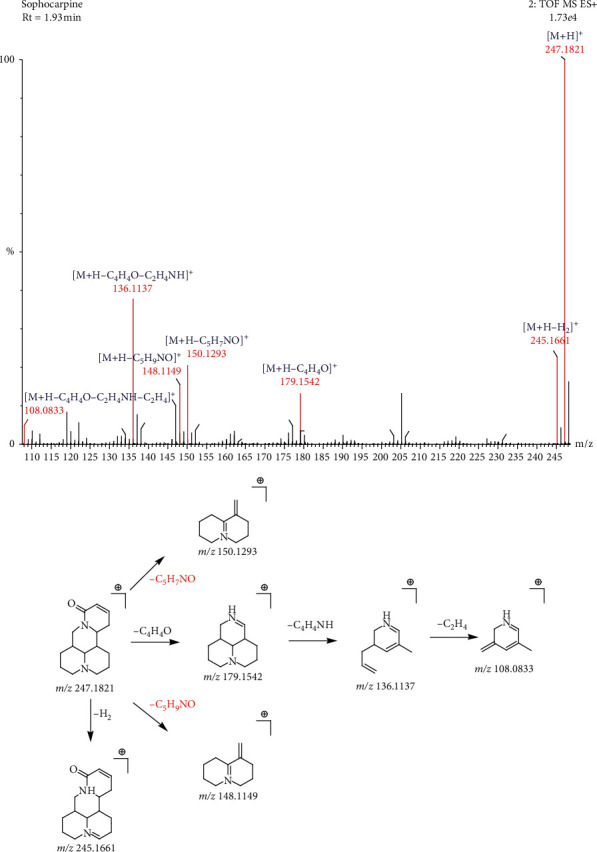
The fragmentation process of sophocarpine in the positive ion mode.

The molecular formula of compound 4 is C_15_H_22_N_2_O, and the retention time is 1.93 min. In the positive ion mode, the molecular ion peak is 247.1821[M+H]^+^, and the main secondary fragments are 245.1661, 179.1542, 150.1293, 148.1149, 136.1137, and 108.0833. It is conjectured that the fragmentation process of compound 4 is as follows. Firstly, the parent ions lose a molecule of C_5_H_7_NO (97) and C_5_H_9_NO (99) to produce the characteristic ion fragments of matrine type: *m/z* 150.1293[M+H-H_2_-C_5_H_7_NO]^+^ and *m/z* 148.1149[M+H-H_2_-C_5_H_9_NO]^+^. Secondly, the parent ion can lose a molecule of C_4_H_4_O resulting in the fragment 179.1542[M+H-C_4_H_4_O]^+^ and then directly lose a molecule of C_2_H_4_NO or lose C_2_H_4_ to yield 136.1137[M+H-C_4_H_4_O-C_2_H_4_NO]^+^ and *m/z* 108.0833[M+H-C_4_H_4_O-C_2_H_4_NO-C_2_H_4_]^+^ ion fragments. The fragment ion 245.1661 is obtained by direct loss of a molecule of H_2_ by the parent ion. Based on the fragmentation rules and standard information, it can be inferred that the compound is sophocarpine. The fragmentation process is shown in Figure 4.

### 3.3. Fragmentation Rules of Flavonoid Compounds

Flavonoids in *Sophora flavescens* mainly include dihydroflavonoids, chalcones, dihydroflavonols, flavonols, and isoflavones, in which dihydroflavonoids and chalcones are easy to change. Therefore, mass spectrometry can well distinguish the two [26]. It is easy to remove neutral molecules from flavonoids such as H_2_O, CH_3_, CO, CO_2_, C_2_H_2_O, and C_2_O_3_ in the negative ion mode. Most of the dihydroflavonoids in *Sophora flavescens* have fragment information such as [M-H]^−^, [M-H-H_2_O]^−^, [M-H-CO]^−^, and [M-H-CH_3_]^−^, and these compounds are prone to RDA cleavage at positions 1,2 and 3,4, resulting in ^1,3^A^−^fragment ions. Chalcone compounds form 261[C_16_H_21_O_3_]^−^ and 161[C_9_H_5_O_3_]^−^ ion fragments under the anion mode B ring, and the 1,4 cleavage occurs in different positions of dihydroflavonoids, resulting in ^1,4^A^−^ and B^1,4−^ characteristic fragment ions [12, 22]. The main fragment of dihydroflavonols is that the C ring is rearranged by RDA to produce characteristic fragments such as 177[C_9_H_5_O_4_]^−^, 275[C_17_H_23_O_3_]^−^, ^1,3^A^−^, and ^1,3^B^−^, and the hydroxyl group at position 3 is unstable, so it is easy to eliminate the reaction and lose H_2_O to form a double bond. Flavonol compounds undergo RDA cleavage to produce characteristic fragments ^1,3^A^−^ and ^1,3^B^−^ and continue to lose neutral molecules such as CO (28) and CO_2_ (44). Isoflavones easily lose neutral molecules such as CO, 2CO, CO_2_, and C_2_O_3_ [23]. Based on the above mass spectrometry information combined with retention time, the chemical constituents of flavonoids in *Sophora flavescens* were identified quickly.

**Figure 5:**
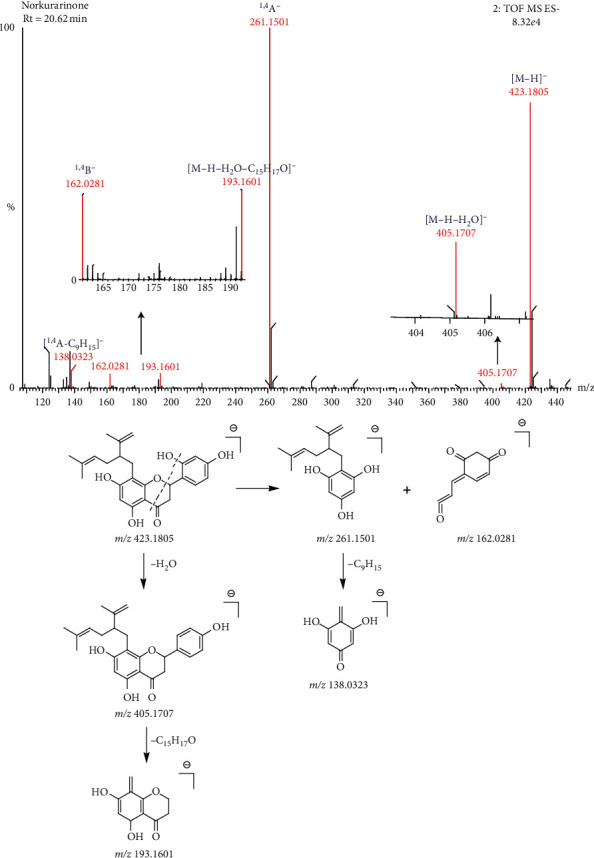
The fragmentation process of norkurarinone in the negative ion mode.

The molecular formula of compound 34 is C_26_H_28_O_6_, and the retention time is 20.62 min. The main secondary fragment ions are 405.1707, 262.15351, 193.1601, 161.0249, and 138.0323. In the negative ion mode, the parent ion peak is *m/z* 423.1805[M-H]^−^. The parent ion 2′-OH is chemically active, which means that it easily loses a molecule of H_2_O and produces dehydrated fragments *m/z* 405.1707[M-H-H_2_O]^−^. On this basis, the neutral losing fragment C_15_H_17_O is lost, and the ion fragment *m/z* 193.1601[M-H-H_2_O-C_15_H_17_O]^−^ is obtained. Due to the presence of 2′-OH, the compound does not easily produce RDA cleavage, but breaks at the 1′4 position of the C ring, resulting in ion fragments ^1,4^A *m/z* 261.1501 and ^1,4^B *m/z* 162.0281, and then the ion fragments *m/z* 138.0323[^1,4^A-C_9_H_15_]^−^ are obtained when C_9_H_15_ is lost in ^1,4^A. Based on the above law, it is inferred that the compound is norkurarinone. The fragmentation process is shown in Figure 5.

**Figure 6:**
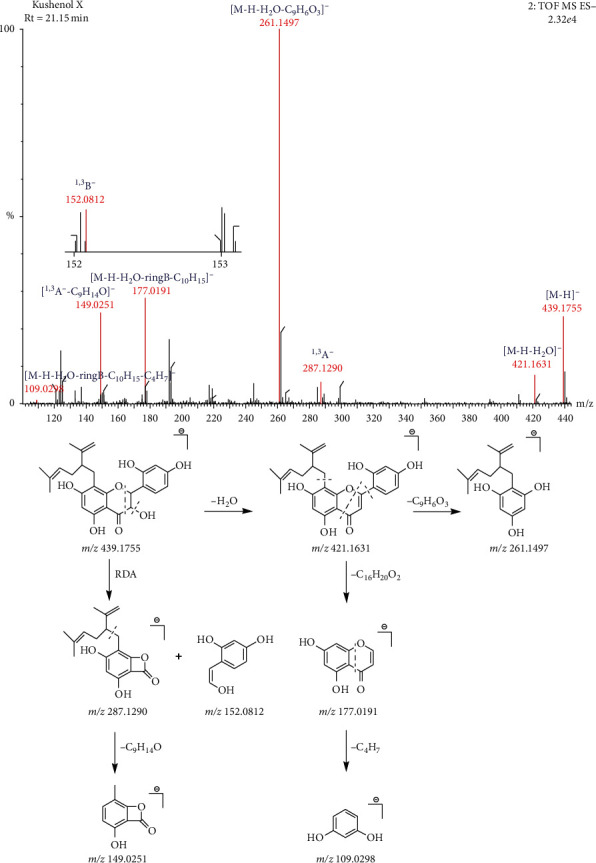
The fragmentation process of kushenol X in the negative ion mode.

The molecular formula of compound 35 is C_25_H_28_O_7_, the retention time is 21.15, and the main secondary fragment ions are 421.1631, 287.12901, 261.1497, 177.0191, 152.08121, 149.0251, and 109.0298. In the negative ion mode, the precursor ion peak is *m/z* 439.1761[M-H]^−^, and the precursor ion removes one molecule of H_2_O to obtain *m/z* 421.1631[M-H-H_2_O]^−^ ion fragment. After that, the 1,4 positions of the C ring break off one molecule of C_9_H_6_O_3_ to obtain the ion fragment 261.1497[M-H-H_2_O-C_9_H_6_O_3_]^−^. In addition, the parent ion of the compound can also directly lose one molecule of C_16_H_20_O_2_, generating ion fragments of *m/z* 177.0197[M-H-H_2_O-ringB-C_10_H_15_]^−^, on the basis of which another molecule of C_4_H_7_ is lost, and *m/z* 109.0298[M-H-H_2_O-ringB-C_10_H_15_-C_4_H_7_]^−^. In the case of RDA rearrangement of this compound, *m/z* 287.1290 ^1,3^A^−^ and *m/z* 152.0812 ^1,3^B^−^ ion fragments can be generated, and then the characteristic fragment ^1,3^A^−^ loses C_9_H_14_O to produce *m/z* 149.0251[^1,3^A^−^-C_9_H_14_O]^−^. Based on the above fragmentation rules, it can be inferred that this compound is kushenol X. The fragmentation process is shown in Figure 6.

## 4. Conclusion

The UHPLC-Q-TOF/MS technique combined with characteristic fragments and neutral loss was applied to the tracking and identification of alkaloids and flavonoids in *Sophora flavescens*, and the fragmentation rules of different parent ions were inferred. A total of 13 alkaloids and 24 flavonoids were identified. Analytical strategies for characterizing the structure of compounds by obtaining diagnostic fragment ions based on excimer ion peaks and MS/MS were summarized.

## Figures and Tables

**Table 1:** Fragmentation information of *Sophora flavescens* in the positive and negative ion mode.

No.	Identity	Formula	RT	Theoretical value	Actual value	ppm	Main MS/MS fragments detected (arranged from large to small according to relative intensity)	Ref.
1	Lamprolobine	C_15_H_24_N_2_O_2_	0.72	265.1916	265.1926	3.77	265.1926[M+H]^+^ 263.1771[M+H-H_2_]^+^ 150.1293[M+H-H_2_O-C_5_H_7_NO]^+^	[12]

2	Matrine	C_15_H_24_N_2_O	1.59	249.1967	249.1980	5.22	249.1980[M+H]^+^ 247.1815[M+H-H_2_]^+^ 150.1298[M+H-H_2_-C_5_H_7_NO]^+^ 148.1152[M+H-H_2_-C_5_H_9_NO]^+^ 136.1144[M+H-H_2_-C_6_H_9_NO]^+^ 176.1083[M+H-H_2_-C_3_H_5_NO]^+^ 231.1959[M+H-H_2_O]^+^	[13, 14]

3	7,11-Dehydromatrine	C_15_H_22_N_2_O	1.93	247.1810	247.1821	4.45	247.1821[M+H]^+^ 148.1149[M+H-C_5_H_9_NO]^+^ 176.1066[M+H-C_3_H_5_NO]^+^	[12]

4	Sophocarpine	C_15_H_22_N_2_O	1.93	247.1810	247.1821	4.45	247.1821[M+H]^+^ 136.1137[M+H C_4_H_4_O-C_2_H_4_NH]^+^ 245.1661[M+H-H_2_]^+^ 150.1293[M+H-C_5_H_7_NO]^+^ 148.1149[M+H-C_5_H_9_NO]^+^ 179.1542[M+H-C_4_H_4_O]^+^ 108.0833[M+H-C_4_H_4_O-C_2_H_4_NH-C_2_H_4_]^+^	[14]

5	Sophoridine	C_15_H_24_N_2_O	1.96	249.1967	249.1983	6.42	249.1983[M+H]^+^ 247.1827[M+H-H_2_]^+^ 150.1294[M+H-H_2_-C_5_H_7_NO]^+^ 148.1146[M+H-H_2_-C_5_H_9_NO]^+^ 218.1494[M+H-CH_5_N]^+^ 231.1847[M+H-H_2_O]^+^	[14]

6	9*α*-Hydroxysophocarpine	C_15_H_22_N_2_O_2_	2.14	263.1760	263.1776	6.08	263.1776[M+H]^+^ 245.1667[M+H-H_2_O]^+^ 150.1299[M+H-H_2_O-C_2_H_2_O-CH_2_-C_2_HN]^+^ 203.1198[M+H-H_2_O-C_2_H_2_O]^+^ 148.1139[M+H-H_2_O-C_2_H_2_O-CH_2_-C_2_HN H_2_]^+^ 175.1250[M+H-H_2_O-C_2_H_2_O-CH_2_-CH_2_]^+^ 189.1109[M+H-H_2_O-C_2_H_2_O-CH_2_]^+^	[15]

7	Mamanine	C_15_H_22_N_2_O_2_	2.14	263.1760	263.1776	6.08	263.1776[M+H]^+^ 150.1299[M+H-H_2_O-C_5_H_5_NO]^+^ 245.1667[M+H-H_2_O]^+^	[12]

8	9*α*-Hydroxymatrine	C_15_H_24_N_2_O_2_	2.29	265.1916	265.1928	4.53	265.1928[M+H]^+^ 150.1294[M+H-H_2_O C_5_H_7_NO]^+^ 247.1824[M+H-H_2_O]^+^	[15]

9	Oxysophoridine	C_15_H_24_N_2_O_2_	2.33	265.1916	265.1933	6.41	265.1933[M+H]^+^ 247.1826[M+H-H_2_O]^+^ 148.1142[M+H-H_2_O-C_5_H_9_NO]^+^ 98.0987[M+H-H_2_O-C_4_H_10_O_2_]^+^ 112.0782[M+H-C_5_H_7_NO-C_2_H_2_NO]^+^ 168.1405[M+H-C_5_H_7_NO]^+^	[12]

10	Oxysophocarpine	C_15_H_22_N_2_O_2_	2.65	263.1760	263.1760	0.00	263.1760[M+H]^+^ 177.1402[M+H-H_2_O-C_4_H_4_O]^+^ 245.1662[M+H-H_2_O]^+^ 150.1289[M+H-C_6_H_11_NO]^+^ 148.1143[M+H-C_5_H_9_NO]^+^ 203.1214[M+H-H_2_O-C_3_H_6_]^+^ 122.1008[M+H-H_2_O-C_4_H_4_O-C_3_H_4_NO]^+^	[12]

11	Oxymatrine	C_15_H_24_N_2_O_2_	3.24	265.1916	265.1926	3.77	265.1926[M+H]^+^ 247.1823[M+H-H_2_O] 205.1355[M+H-H_2_O-C_2_H_4_N]+ 148.1139[M+H-H_2_O-C_5_H_9_NO]+ 175.1254[M+H-C_4_H_8_O]+ 112.1129[M+H-C_5_H_7_NO-C_2_H_2_NO]+	[13, 14]

12	Sophoranol N-oxide	C_15_H_24_N_2_O_3_	4.68	281.1865	281.1879	4.98	281.1879[M+H]^+^ 245.1696[M+H-2H_2_O]^+^ 138.1283[M+H-H_2_O-C_4_H_10_NO_2_]^+^ 263.1678[M+H-H_2_O]^+^	[15]

13	Daidzin	C_21_H_20_O_9_	6.19	417.1186	417.1184	−0.48	417.1184[M+H]^+^ 255.0589[M+H-Glu]^+^ 199.0759[M+H-Glu-2CO]^+^	[12, 16]

14	Baptifoline	C_15_H_20_N_2_O_2_	6.70	261.1603	261.1595	−3.06	243.1435[M+H-H_2_O]^+^ 261.1595[M+H]^+^ 146.1015[M+H-C_6_H_13_NO]^+^ 96.0877[M+H-H_2_O-C_9_H_9_NO]^+^ 114.0939[M+H-C_9_H_9_NO]^+^	[16]

15	Daidzein	C_15_H_10_O_4_	11.03	255.0657	255.0690	12.94	255.0690[M+H-Glu]^+^ 137.0274[M+H-C_2_O_3_]^+^ 199.0759[M+H-Glu-2CO]^+^	[16]

16	Kurarinone	C_26_H_30_O_6_	19.43	439.2121	439.2128	1.59	439.2128[M+H]^+^ 179.0361[^1,3^A^+^-C_9_H_16_]^+^ 303.1609^1,3^A^+^ 136.0179^1,3^B^+^ (136.0179^1, 3^B^+^ is the fragment ion produced by RDA fragmentation reaction) 421.2132[M+H-H_2_O]^+^	[17, 18]

17	Stamens isoflavones	C_16_H_12_O_5_	12.26	283.0606	283.0618	4.24	283.0616[M-H]^−^ 268.0373[M-H-CH_3_]^−^ 211.0400[M-H-C_2_O_3_]^−^ 253.0472[M-H-3H_2_O]^−^ 271.1014[M-H-H_2_O]^−^	[12, 19]

18	(2R,3R)-8-Isopentenyl-7,4-dihydroxy-5-methoxy dihydroflavonol	C_21_H_22_O_6_	16.04	369.1338	369.1349	2.98	369.1349[M-H]^−^ 313.0851[M-H-C_3_H_4_O]^−^ 353.9778[M-H-CH_3_]^−^ 351.1122[M-H-H_2_O]^−^	[12]

19	Formononetin	C_16_H_12_O_4_	16.29	267.0657	267.0669	4.49	267.0669[M-H]^−^ 252.0430[M-H-CH_3_]^−^ 223.0404[M-H-CO_2_]^−^	[20]

20	2′-Hydroxy-isoxanthohumol	C_21_H_22_O_6_	16.58	369.1338	369.1346	2.17	369.1346[M-H]^−^ 207.1024[M-H-H_2_O-C_8_H_16_O_2_]^−^ 341.1375[M-H-CO]^−^ 351.1252[M-H-H_2_O]^−^ 354.4461[M-H-CH_3_]^−^	[12]

21	Kuraridinol	C_26_H_32_O_7_	16.68	455.2070	455.2076	1.32	455.2076[M-H]^−^ 161.0246[C_9_H_5_O_3_]^−^ 293.1761^1,4^A^−^ 437.1793[M-H-H_2_O]^−^	[19, 21]

22	Leachianone G	C_20_H_20_O_6_	17.37	355.1182	355.1202	5.63	355.1202[M-H]^−^ 161.0214[C_9_H_5_O_3_]^−^ 337.1068[M-H-H_2_O]^−^ 235.1366^1,3^A^−^	[17]

23	Norkurarinol	C_25_H_30_O_7_	18.24	441.1913	441.1928	3.40	279.1612^1,3^A^−^ 441.1928[M-H]^−^ 161.0251[C_9_H_5_O_3_]^−^ 211.1704[^1,3^A^−^-C_3_O_2_]^−^ 162.0280^1,3^B^−^ 423.1707[M-H-H_2_O]^−^	[21]

24	Kushenol Q	C_25_H_28_O_11_	18.39	441.1913	441.1935	4.99	279.1617^1,3^A^−^441.1935[M-H]^−^ 161.0255[C_9_H_5_O_3_]^−^ 211.1747[^1,3^A^−^-C_3_O_2_]^−^ 331.1563[M-H-C_8_H_14_]^−^	[22]

25	Maackiain	C_16_H_12_O_5_	18.48	283.0606	283.0598	−2.83	283.0598[M-H]^−^ 268.0368[M-H-CH_3_]^−^ 211.0394[M-H-C_2_O_3_]^−^ 227.0754[M-H-2CO]^−^ 255.0628[M-H-CO]^−^	[16]

26	Kushenol N	C_26_H_30_O_7_	18.76	453.1913	453.1913	0.00	453.1913[M-H]^−^ 177.0195[C_9_H_5_O_4_]^−1,4^B^−^ 275.1653[C_17_H_23_O_3_]^−1,4^A^−^ 149.0249[^1,4^B^−^-CO]^−^ 137.0254[^1,4A−^-C_9_H_15_-CH_3_]^−^	[23]

27	Kushenol I	C_26_H_30_O_7_	19.04	453.1913	453.1908	−1.10	453.1908[M-H]^−^ 177.0191[C_9_H_5_O_4_]^−1,4^B^−^ 149.0256[^1,4^B^−^-CO]^−^ 435.1866[M-H-H_2_O]^−^ 425.2036[M-H-CO]^−^	[16]

28	Sophoraflavanone B	C_20_H_20_O_5_	19.08	339.1232	339.1231	−0.29	219.0664^1,4^A^−^ 339.1231[M-H]^−^ 119.0504[^1,4^A-C_7_H_16_]^−^ 275.1648[M-H-C_4_H_4_O]^−^ 321.9993[M-H-H_2_O]^−^	[16]

29	Noranhydroicaritin	C_20_H_18_O_6_	19.17	353.1025	353.1034	2.55	353.1034[M-H]^−^ 298.0487[M-H-C_4_H_7_]^−^ 136.0176[M-H-C_4_H_7_-C_9_H_22_O_2_]^−^ 338.0812[M-H-CH_3_]^−^ 161.0250[C_9_H_5_O_3_]^−^	[24]

30	Kushenol L	C_25_H_28_O_7_	19.43	439.1757	439.1760	0.68	439.1760[M-H]^−^ 275.1653[C_17_H_23_O_3_]^−^ 177.0201[C_9_H_5_O_4_]^−^ 149.0247[M-H-C_9_H_14_O]^−^ 421.1667[M-H-H_2_O]^−^	[24]

31	Sophoraisoflavanone A	C_21_H_22_O_6_	20.18	369.1338	369.1360	5.96	161.0255^1,4^B^−^ 369.1360[M-H]^−^ 135.0452[^1,4^B^−^-CH_2_]^−^ 275.1674[M-H-C_8_H_10_]^−^ 208.1087^1,4^A^−^	[12]

32	8-Lavandulylkaempferol	C_26_H_30_O_5_	20.39	421.2015	421.2027	2.85	421.2027[M-H]^−^ 301.1454^1,4^A^−^ 119.0511[^1,4^A^−^-C_9_H_14_O-C_3_H_8_]^−^ 163.0040[^1,4^A^−^-C_9_H_14_O]^−^	[12, 16]

33	Kushenol D	C_27_H_32_O_6_	20.56	451.2121	451.2118	−0.66	451.2118[M-H]^−^ 301.1442^1,4^A^−^ 149.0617^1,4^B^−^ 217.0517[^1,4^A^−^-C_6_H_12_]^−^ 419.1871[M-H-C_2_O_2_]^−^	[24]

34	Norkurarinone	C_25_H_28_O_6_	20.62	423.1808	423.1805	−0.71	423.1805[M-H]^−^ 161.0249^1,4^B^−^ 262.1535^1,4^A^−^ 138.0323[^1,4^A^−^-C_9_H_15_]^−^ 193.1601[M-H-H_2_O-C_15_H_17_O]^−^ 405.1707[M-H-H_2_O]^−^	[17]

35	Kushenol X	C_25_H_28_O_7_	21.15	439.1757	439.1755	−0.46	261.1497[M-H-H_2_O-ringB-C_10_H_15_]^−^ 177.0191[M-H-H_2_O-ringB-C_10_H_15_]^−^ 149.0251[M-H-C_9_H_14_O]^−^ 439.1755[M-H]^−^ 421.1631[M-H-H_2_O]^−^ 287.1290^1,3^A^−^ 109.0298[M-H-H_2_O-ringB-C_10_H_15_-C_4_H_7_]^−^ 152.0812^1,3^B^−^	[12, 22]

36	Kuraridin	C_26_H_30_O_6_	23.04	437.1964	437.1984	4.57	161.0258^1,4^B^−^ 275.1667^1,4^A^−^ 437.1984[M-H]^−^ 151.0412[^1,4^A^−^-C_9_H_16_]^−^	[23]

37	5-Methylkushenol C	C_27_H_32_O_6_	24.61	451.2121	451.2141	4.43	451.2141[M-H]^−^ 301.1473^1,3^A^−^ 192.0471[^1,3^A^−^-C_8_H_13_]^−^ 313.0871[M-H-C_6_H_12_-C_4_H_6_]^−^ 367.1223[M-H-C_6_H_12_]^−^	[12, 21]

## Data Availability

No data were used to support this study.
